# Supplementary material for: Pyruvate Dehydrogenase Kinase 1 inhibition mediated oxidative phosphorylation enhancement in cartilage promotes osteoarthritis progression
Source: BMC Musculoskelet Disord. 2023 Jul 20;24:597. doi: 10.1186/s12891-023-06585-6 (PMC10357736; doi:10.1186/s12891-023-06585-6)
Supplement: Supplementary file 1 — Additional file 1. [file 12891_2023_6585_MOESM1_ESM.zip › BP.Gsea.1653623667859/GOBP_POSITIVE_REGULATION_OF_MRNA_METABOLIC_PROCESS.html]

Details for gene set GOBP\_POSITIVE\_REGULATION\_OF\_MRNA\_METABOLIC\_PROCESS[GSEA]

|  || Dataset | 55457\_collapsed\_to\_symbols.cls.cls #OA\_versus\_normal.cls.cls #OA\_versus\_normal\_repos |
| Phenotype | cls.cls#OA\_versus\_normal\_repos |
| Upregulated in class | normal |
| GeneSet | GOBP\_POSITIVE\_REGULATION\_OF\_MRNA\_METABOLIC\_PROCESS |
| Enrichment Score (ES) | -0.41121942 |
| Normalized Enrichment Score (NES) | -1.765502 |
| Nominal p-value | 0.0 |
| FDR q-value | 0.11736706 |
| FWER p-Value | 0.998 |
Table: GSEA Results Summary

  

Fig 1: Enrichment plot: GOBP\_POSITIVE\_REGULATION\_OF\_MRNA\_METABOLIC\_PROCESS      
 Profile of the Running ES Score & Positions of GeneSet Members on the Rank Ordered List

  

| SYMBOL | TITLE | RANK IN GENE LIST | RANK METRIC SCORE | RUNNING ES | CORE ENRICHMENT || 1 | POLR2G | RNA polymerase II subunit G [Source:HGNC Symbol;Acc:HGNC:9194] | 183 | 0.830 | 0.0117 | No |
| 2 | PAN2 | poly(A) specific ribonuclease subunit PAN2 [Source:HGNC Symbol;Acc:HGNC:20074] | 190 | 0.828 | 0.0372 | No |
| 3 | MLH1 | mutL homolog 1 [Source:HGNC Symbol;Acc:HGNC:7127] | 497 | 0.660 | 0.0340 | No |
| 4 | PRR5L | proline rich 5 like [Source:HGNC Symbol;Acc:HGNC:25878] | 1198 | 0.477 | -0.0056 | No |
| 5 | FTO | FTO alpha-ketoglutarate dependent dioxygenase [Source:HGNC Symbol;Acc:HGNC:24678] | 1314 | 0.456 | -0.0003 | No |
| 6 | LSM1 | "LSM1 homolog, mRNA degradation associated [Source:HGNC Symbol;Acc:HGNC:20472]" | 1470 | 0.433 | 0.0011 | No |
| 7 | CNOT7 | CCR4-NOT transcription complex subunit 7 [Source:HGNC Symbol;Acc:HGNC:14101] | 1676 | 0.402 | -0.0023 | No |
| 8 | NUDT21 | nudix hydrolase 21 [Source:HGNC Symbol;Acc:HGNC:13870] | 1734 | 0.393 | 0.0056 | No |
| 9 | EXOSC7 | exosome component 7 [Source:HGNC Symbol;Acc:HGNC:28112] | 1969 | 0.364 | -0.0012 | No |
| 10 | DCPS | "decapping enzyme, scavenger [Source:HGNC Symbol;Acc:HGNC:29812]" | 2570 | 0.295 | -0.0388 | No |
| 11 | DCP2 | decapping mRNA 2 [Source:HGNC Symbol;Acc:HGNC:24452] | 2659 | 0.284 | -0.0368 | No |
| 12 | PARN | poly(A)-specific ribonuclease [Source:HGNC Symbol;Acc:HGNC:8609] | 3100 | 0.240 | -0.0636 | No |
| 13 | CIRBP | cold inducible RNA binding protein [Source:HGNC Symbol;Acc:HGNC:1982] | 3441 | 0.207 | -0.0836 | No |
| 14 | RIDA | reactive intermediate imine deaminase A homolog [Source:HGNC Symbol;Acc:HGNC:16897] | 3488 | 0.203 | -0.0809 | No |
| 15 | CNOT1 | CCR4-NOT transcription complex subunit 1 [Source:HGNC Symbol;Acc:HGNC:7877] | 3842 | 0.172 | -0.1030 | No |
| 16 | ZC3HAV1 | "zinc finger CCCH-type containing, antiviral 1 [Source:HGNC Symbol;Acc:HGNC:23721]" | 3946 | 0.161 | -0.1060 | No |
| 17 | DIS3 | "DIS3 homolog, exosome endoribonuclease and 3'-5' exoribonuclease [Source:HGNC Symbol;Acc:HGNC:20604]" | 4187 | 0.139 | -0.1204 | No |
| 18 | EXOSC5 | exosome component 5 [Source:HGNC Symbol;Acc:HGNC:24662] | 4295 | 0.128 | -0.1247 | No |
| 19 | YTHDF1 | YTH N6-methyladenosine RNA binding protein 1 [Source:HGNC Symbol;Acc:HGNC:15867] | 4320 | 0.127 | -0.1226 | No |
| 20 | UPF1 | UPF1 RNA helicase and ATPase [Source:HGNC Symbol;Acc:HGNC:9962] | 4434 | 0.118 | -0.1277 | No |
| 21 | CNOT8 | CCR4-NOT transcription complex subunit 8 [Source:HGNC Symbol;Acc:HGNC:9207] | 4581 | 0.106 | -0.1358 | No |
| 22 | HNRNPD | heterogeneous nuclear ribonucleoprotein D [Source:HGNC Symbol;Acc:HGNC:5036] | 4830 | 0.085 | -0.1525 | No |
| 23 | PAIP1 | poly(A) binding protein interacting protein 1 [Source:HGNC Symbol;Acc:HGNC:16945] | 4851 | 0.083 | -0.1515 | No |
| 24 | EIF4ENIF1 | eukaryotic translation initiation factor 4E nuclear import factor 1 [Source:HGNC Symbol;Acc:HGNC:16687] | 5330 | 0.045 | -0.1873 | No |
| 25 | SNW1 | SNW domain containing 1 [Source:HGNC Symbol;Acc:HGNC:16696] | 5472 | 0.035 | -0.1973 | No |
| 26 | CDC73 | cell division cycle 73 [Source:HGNC Symbol;Acc:HGNC:16783] | 5604 | 0.023 | -0.2067 | No |
| 27 | CNOT2 | CCR4-NOT transcription complex subunit 2 [Source:HGNC Symbol;Acc:HGNC:7878] | 5740 | 0.012 | -0.2169 | No |
| 28 | PAF1 | "PAF1 homolog, Paf1/RNA polymerase II complex component [Source:HGNC Symbol;Acc:HGNC:25459]" | 5901 | 0.002 | -0.2293 | No |
| 29 | GIGYF2 | GRB10 interacting GYF protein 2 [Source:HGNC Symbol;Acc:HGNC:11960] | 6123 | -0.014 | -0.2461 | No |
| 30 | CNOT3 | CCR4-NOT transcription complex subunit 3 [Source:HGNC Symbol;Acc:HGNC:7879] | 6183 | -0.019 | -0.2501 | No |
| 31 | CPEB1 | cytoplasmic polyadenylation element binding protein 1 [Source:HGNC Symbol;Acc:HGNC:21744] | 6435 | -0.038 | -0.2685 | No |
| 32 | METTL3 | "methyltransferase 3, N6-adenosine-methyltransferase complex catalytic subunit [Source:HGNC Symbol;Acc:HGNC:17563]" | 6685 | -0.055 | -0.2863 | No |
| 33 | SF3B4 | splicing factor 3b subunit 4 [Source:HGNC Symbol;Acc:HGNC:10771] | 6777 | -0.061 | -0.2915 | No |
| 34 | PRDX6 | peroxiredoxin 6 [Source:HGNC Symbol;Acc:HGNC:16753] | 6783 | -0.061 | -0.2899 | No |
| 35 | DCP1A | decapping mRNA 1A [Source:HGNC Symbol;Acc:HGNC:18714] | 7525 | -0.121 | -0.3440 | No |
| 36 | MEX3D | mex-3 RNA binding family member D [Source:HGNC Symbol;Acc:HGNC:16734] | 7652 | -0.131 | -0.3497 | No |
| 37 | POLR2D | RNA polymerase II subunit D [Source:HGNC Symbol;Acc:HGNC:9191] | 7726 | -0.136 | -0.3512 | No |
| 38 | TARDBP | TAR DNA binding protein [Source:HGNC Symbol;Acc:HGNC:11571] | 7864 | -0.146 | -0.3573 | No |
| 39 | TOB1 | "transducer of ERBB2, 1 [Source:HGNC Symbol;Acc:HGNC:11979]" | 7892 | -0.147 | -0.3548 | No |
| 40 | CPEB3 | cytoplasmic polyadenylation element binding protein 3 [Source:HGNC Symbol;Acc:HGNC:21746] | 8060 | -0.160 | -0.3628 | No |
| 41 | PDE12 | phosphodiesterase 12 [Source:HGNC Symbol;Acc:HGNC:25386] | 8306 | -0.178 | -0.3763 | No |
| 42 | NCBP2 | nuclear cap binding protein subunit 2 [Source:HGNC Symbol;Acc:HGNC:7659] | 8478 | -0.192 | -0.3837 | No |
| 43 | EXOSC8 | exosome component 8 [Source:HGNC Symbol;Acc:HGNC:17035] | 8554 | -0.198 | -0.3833 | No |
| 44 | TUT7 | terminal uridylyl transferase 7 [Source:HGNC Symbol;Acc:HGNC:25817] | 8629 | -0.203 | -0.3827 | No |
| 45 | RBM3 | RNA binding motif protein 3 [Source:HGNC Symbol;Acc:HGNC:9900] | 8859 | -0.222 | -0.3937 | No |
| 46 | RBMX | RNA binding motif protein X-linked [Source:HGNC Symbol;Acc:HGNC:9910] | 8938 | -0.227 | -0.3926 | No |
| 47 | GTPBP1 | GTP binding protein 1 [Source:HGNC Symbol;Acc:HGNC:4669] | 8950 | -0.228 | -0.3864 | No |
| 48 | SAMD4A | sterile alpha motif domain containing 4A [Source:HGNC Symbol;Acc:HGNC:23023] | 9027 | -0.234 | -0.3849 | No |
| 49 | ZC3H12A | zinc finger CCCH-type containing 12A [Source:HGNC Symbol;Acc:HGNC:26259] | 9030 | -0.234 | -0.3778 | No |
| 50 | HSPA8 | heat shock protein family A (Hsp70) member 8 [Source:HGNC Symbol;Acc:HGNC:5241] | 9140 | -0.243 | -0.3786 | No |
| 51 | NCBP1 | nuclear cap binding protein subunit 1 [Source:HGNC Symbol;Acc:HGNC:7658] | 9149 | -0.244 | -0.3716 | No |
| 52 | TRA2B | transformer 2 beta homolog [Source:HGNC Symbol;Acc:HGNC:10781] | 9243 | -0.251 | -0.3710 | No |
| 53 | YTHDF3 | YTH N6-methyladenosine RNA binding protein 3 [Source:HGNC Symbol;Acc:HGNC:26465] | 9549 | -0.278 | -0.3861 | No |
| 54 | YBX1 | Y-box binding protein 1 [Source:HGNC Symbol;Acc:HGNC:8014] | 9623 | -0.285 | -0.3829 | No |
| 55 | HNRNPU | heterogeneous nuclear ribonucleoprotein U [Source:HGNC Symbol;Acc:HGNC:5048] | 9847 | -0.306 | -0.3907 | No |
| 56 | TENT4A | terminal nucleotidyltransferase 4A [Source:HGNC Symbol;Acc:HGNC:16705] | 10003 | -0.322 | -0.3927 | No |
| 57 | PUM1 | pumilio RNA binding family member 1 [Source:HGNC Symbol;Acc:HGNC:14957] | 10145 | -0.335 | -0.3932 | No |
| 58 | TUT4 | terminal uridylyl transferase 4 [Source:HGNC Symbol;Acc:HGNC:28981] | 10377 | -0.359 | -0.4000 | Yes |
| 59 | ZFP36L2 | ZFP36 ring finger protein like 2 [Source:HGNC Symbol;Acc:HGNC:1108] | 10509 | -0.374 | -0.3985 | Yes |
| 60 | CSDE1 | cold shock domain containing E1 [Source:HGNC Symbol;Acc:HGNC:29905] | 10559 | -0.379 | -0.3904 | Yes |
| 61 | EXOSC9 | exosome component 9 [Source:HGNC Symbol;Acc:HGNC:9137] | 10606 | -0.384 | -0.3819 | Yes |
| 62 | DHX9 | DExH-box helicase 9 [Source:HGNC Symbol;Acc:HGNC:2750] | 10627 | -0.386 | -0.3714 | Yes |
| 63 | ROCK2 | Rho associated coiled-coil containing protein kinase 2 [Source:HGNC Symbol;Acc:HGNC:10252] | 10648 | -0.388 | -0.3608 | Yes |
| 64 | NUP98 | nucleoporin 98 and 96 precursor [Source:HGNC Symbol;Acc:HGNC:8068] | 10875 | -0.413 | -0.3655 | Yes |
| 65 | RC3H2 | ring finger and CCCH-type domains 2 [Source:HGNC Symbol;Acc:HGNC:21461] | 11102 | -0.444 | -0.3692 | Yes |
| 66 | SYNCRIP | synaptotagmin binding cytoplasmic RNA interacting protein [Source:HGNC Symbol;Acc:HGNC:16918] | 11182 | -0.454 | -0.3612 | Yes |
| 67 | HSF1 | heat shock transcription factor 1 [Source:HGNC Symbol;Acc:HGNC:5224] | 11189 | -0.456 | -0.3473 | Yes |
| 68 | CNOT6 | CCR4-NOT transcription complex subunit 6 [Source:HGNC Symbol;Acc:HGNC:14099] | 11212 | -0.459 | -0.3347 | Yes |
| 69 | AGO2 | argonaute RISC catalytic component 2 [Source:HGNC Symbol;Acc:HGNC:3263] | 11246 | -0.463 | -0.3227 | Yes |
| 70 | PRPF19 | pre-mRNA processing factor 19 [Source:HGNC Symbol;Acc:HGNC:17896] | 11269 | -0.467 | -0.3098 | Yes |
| 71 | KHSRP | KH-type splicing regulatory protein [Source:HGNC Symbol;Acc:HGNC:6316] | 11285 | -0.469 | -0.2963 | Yes |
| 72 | METTL16 | "methyltransferase 16, N6-methyladenosine [Source:HGNC Symbol;Acc:HGNC:28484]" | 11346 | -0.478 | -0.2860 | Yes |
| 73 | CELF3 | CUGBP Elav-like family member 3 [Source:HGNC Symbol;Acc:HGNC:11967] | 11510 | -0.502 | -0.2829 | Yes |
| 74 | NOCT | nocturnin [Source:HGNC Symbol;Acc:HGNC:14254] | 11566 | -0.510 | -0.2713 | Yes |
| 75 | BTG2 | BTG anti-proliferation factor 2 [Source:HGNC Symbol;Acc:HGNC:1131] | 11917 | -0.576 | -0.2805 | Yes |
| 76 | TNRC6B | trinucleotide repeat containing adaptor 6B [Source:HGNC Symbol;Acc:HGNC:29190] | 11947 | -0.582 | -0.2645 | Yes |
| 77 | ROCK1 | Rho associated coiled-coil containing protein kinase 1 [Source:HGNC Symbol;Acc:HGNC:10251] | 12046 | -0.605 | -0.2532 | Yes |
| 78 | SNRNP70 | small nuclear ribonucleoprotein U1 subunit 70 [Source:HGNC Symbol;Acc:HGNC:11150] | 12073 | -0.613 | -0.2360 | Yes |
| 79 | EXOSC2 | exosome component 2 [Source:HGNC Symbol;Acc:HGNC:17097] | 12100 | -0.620 | -0.2186 | Yes |
| 80 | SAMD4B | sterile alpha motif domain containing 4B [Source:HGNC Symbol;Acc:HGNC:25492] | 12247 | -0.659 | -0.2093 | Yes |
| 81 | NCL | nucleolin [Source:HGNC Symbol;Acc:HGNC:7667] | 12255 | -0.662 | -0.1891 | Yes |
| 82 | DND1 | DND microRNA-mediated repression inhibitor 1 [Source:HGNC Symbol;Acc:HGNC:23799] | 12266 | -0.667 | -0.1690 | Yes |
| 83 | CELF1 | CUGBP Elav-like family member 1 [Source:HGNC Symbol;Acc:HGNC:2549] | 12391 | -0.704 | -0.1566 | Yes |
| 84 | THRAP3 | thyroid hormone receptor associated protein 3 [Source:HGNC Symbol;Acc:HGNC:22964] | 12426 | -0.717 | -0.1368 | Yes |
| 85 | TRA2A | transformer 2 alpha homolog [Source:HGNC Symbol;Acc:HGNC:16645] | 12479 | -0.740 | -0.1176 | Yes |
| 86 | PABPC1 | poly(A) binding protein cytoplasmic 1 [Source:HGNC Symbol;Acc:HGNC:8554] | 12562 | -0.778 | -0.0996 | Yes |
| 87 | DAZAP1 | DAZ associated protein 1 [Source:HGNC Symbol;Acc:HGNC:2683] | 12686 | -0.854 | -0.0825 | Yes |
| 88 | YTHDF2 | YTH N6-methyladenosine RNA binding protein 2 [Source:HGNC Symbol;Acc:HGNC:31675] | 12694 | -0.860 | -0.0560 | Yes |
| 89 | ZFP36 | ZFP36 ring finger protein [Source:HGNC Symbol;Acc:HGNC:12862] | 12846 | -1.052 | -0.0349 | Yes |
| 90 | ZFP36L1 | ZFP36 ring finger protein like 1 [Source:HGNC Symbol;Acc:HGNC:1107] | 12888 | -1.244 | 0.0009 | Yes |
Table: GSEA details [plain text format]

  

Fig 2: GOBP\_POSITIVE\_REGULATION\_OF\_MRNA\_METABOLIC\_PROCESS      
 Blue-Pink O' Gram in the Space of the Analyzed GeneSet

  

Fig 3: GOBP\_POSITIVE\_REGULATION\_OF\_MRNA\_METABOLIC\_PROCESS: Random ES distribution      
 Gene set null distribution of ES for **GOBP\_POSITIVE\_REGULATION\_OF\_MRNA\_METABOLIC\_PROCESS**
